# Supplementary material for: From sequence to enzyme mechanism using multi-label machine learning
Source: BMC Bioinformatics. 2014 May 19;15:150. doi: 10.1186/1471-2105-15-150 (PMC4229970; doi:10.1186/1471-2105-15-150)
Supplement: Additional file 2 — Java code of ml2db. Additional file ml2db_code.tar.gz contains the Java source code to run the multi-label machine learning experiments and save the results to database. The code’s Javadoc is included. [file 1471-2105-15-150-S2.zip › additional file 2/ml2db/ml2db/doc/mulan/evaluation/measure/MeasureBase.html]

MeasureBase


JavaScript is disabled on your browser.


- Overview
- Package
- Class
- Use
- Tree
- Deprecated
- Index
- Help

- Prev Class
- Next Class

- Frames
- No Frames

- All Classes

- Summary:
- Nested |
- Field |
- Constr |
- Method

- Detail:
- Field |
- Constr |
- Method


mulan.evaluation.measure

## Class MeasureBase

- java.lang.Object
- - mulan.evaluation.measure.MeasureBase

- All Implemented Interfaces:
  :   java.io.Serializable, Measure

  Direct Known Subclasses:
  :   BipartitionMeasureBase, ConfidenceMeasureBase, RankingMeasureBase

  ---

    

  ```
  public abstract class MeasureBase
  extends java.lang.Object
  implements Measure, java.io.Serializable
  ```

  Author:
  :   Grigorios Tsoumakas

  See Also:
  :   Serialized Form

- - ### Constructor Summary

    Constructors

    Constructor and Description

    `MeasureBase()`
  - ### Method Summary

    Methods

    | Modifier and Type | Method and Description |
    | --- | --- |
    | `Measure` | `makeCopy()` Creates a deep copy of the given measure using serialization. |
    | `java.lang.String` | `toString()` Returns a string with the value of a measure |
    | `void` | `update(MultiLabelOutput prediction, boolean[] truth)` Computes the value of a measure for the given prediction and true labels. |

    - ### Methods inherited from class java.lang.Object

      `equals, getClass, hashCode, notify, notifyAll, wait, wait, wait`
    - ### Methods inherited from interface mulan.evaluation.measure.Measure

      `getIdealValue, getName, getValue, reset`

- - ### Constructor Detail


    - #### MeasureBase

      ```
      public MeasureBase()
      ```
  - ### Method Detail


    - #### update

      ```
      public final void update(MultiLabelOutput prediction,
                boolean[] truth)
      ```

      **Description copied from interface: `Measure`**

      Computes the value of a measure for the given prediction and true labels. The immediate value of
      a measure is returned and result is added to the cumulated measure value.

      **Specified by:**
      :   `update` in interface `Measure`

      Parameters:
      :   `prediction` - the prediction for which measure has to be computed
      :   `truth` - the true labels bipartition for given prediction

      See Also:
      :   `Measure.getValue()`


    - #### toString

      ```
      public java.lang.String toString()
      ```

      Returns a string with the value of a measure

      **Overrides:**
      :   `toString` in class `java.lang.Object`

      Returns:
      :   string representation of the value of a measure


    - #### makeCopy

      ```
      public Measure makeCopy()
                       throws java.lang.Exception
      ```

      **Description copied from interface: `Measure`**

      Creates a deep copy of the given measure using serialization.

      **Specified by:**
      :   `makeCopy` in interface `Measure`

      Returns:
      :   a deep copy of the measure

      Throws:
      :   `java.lang.Exception` - if an error occurs while making copy of the measure.


- Overview
- Package
- Class
- Use
- Tree
- Deprecated
- Index
- Help

- Prev Class
- Next Class

- Frames
- No Frames

- All Classes

- Summary:
- Nested |
- Field |
- Constr |
- Method

- Detail:
- Field |
- Constr |
- Method
